# A national database propensity score-matched comparison of minimally invasive and open colectomy for long-term opioid use

**DOI:** 10.1007/s00464-021-08338-9

**Published:** 2021-02-10

**Authors:** Amir L. Bastawrous, Kara K. Brockhaus, Melissa I. Chang, Gediwon Milky, I.-Fan Shih, Yanli Li, Robert K. Cleary

**Affiliations:** 1 Swedish Cancer Institute, Seattle, WA USA; 2 Inpatient Pharmacy, St. Joseph Mercy Hospital Ann Arbor, Ann Arbor, MI USA; 3 Department of Surgery, St. Joseph Mercy Hospital Ann Arbor, 5325 Elliott Dr. Suite 104, Ann Arbor, MI 48106, USA; 4 Global Health Economics and Outcomes Research, Intuitive Surgical, Inc., Sunnyvale, CA USA; 5 Department of Pharmacy Practice, Purdue University, West Lafayette, IN USA

**Keywords:** Colon resection, Opioid, Minimally invasive, Robotic-assisted surgery, Laparoscopic surgery

## Abstract

**Background:**

Opioid dependence is a public health crisis and surgery is a risk factor for long-term opioid use. Though minimally invasive surgery (MIS) is associated with less perioperative pain, demonstrating an association with less long-term opioid use would be another reason to justify adoption of minimally invasive techniques. We compared the rates for long-term opioid prescriptions among patients in a large national database who underwent minimally invasive and open colectomy.

**Methods:**

Using the MarketScan Database, we retrospectively analyzed patients undergoing colon resection for benign and malignant diseases between 2013 and 2017. Among opioid-naïve patients who had ≥ 1 opioid prescriptions filled perioperatively (30 days before surgery to 14 days after discharge), propensity score matching was applied for group comparisons [open (OS) versus MIS, and laparoscopic (LS) versus robotic-assisted surgery (RS)]. The primary outcome was long-term opioid use defined as the proportion of patients with ≥ 1 long-term opioid prescriptions filled 90–180 days after discharge. Risks factors for long-term opioid use were assessed using logistic regression.

**Results:**

Among the 5413 matched pairs in the MIS versus OS cohorts, MIS significantly reduced long-term opioid use of ‘any opioids’ (13.3% vs. 20.9%), schedule II/III opioids (11.7% vs. 19.2%), and high-dose opioids (4.3% vs. 7.7%; all *p* < 0.001). Among the 1195 matched pairs in the RS versus LS cohorts, RS was associated with less high-dose opioids (2.1% vs. 3.8%, *p* = 0.015) 90–180 days after discharge. Other risk factors for long-term opioid use included younger age, benign indications, tobacco use, mental health conditions, and > 6 Charlson comorbidities.

**Conclusion:**

Minimally invasive colectomy is associated with a significant reduction in long-term opioid use when compared to OS. Robotic-assisted colectomy was associated with less high-dose opioids compared to LS. Increasing adoption of minimally invasive surgery for colectomy and including RS, where appropriate, may decrease long-term opioid use.

**Supplementary Information:**

The online version contains supplementary material available at 10.1007/s00464-021-08338-9.

Opioid dependence and overdose have recently attracted public awareness and concern as a public health problem. In 2017, opioids caused 14.9 overdose deaths per 100,000 United States (US) population. Of these, prescription opioids were associated with 5.2 overdose deaths per 100,000 US population [1]. The US societal economic burden of prescription opioid overdose, abuse and dependence is estimated to be $78.5 billion annually [2].

Short-term opioid use for management of postoperative pain is an important risk factor for long-term opioid use and dependence in major and minor surgeries [3]. A database analysis of seven surgical procedures showed that the proportion of new persistent opioid users was highest among patients who had colectomy (17%), followed by patients who had total knee replacement (15.2%) [4]. If minimally invasive surgery (MIS) is associated with less long-term opioid use than open surgery, then increasing minimally invasive colectomy training efforts and adoption would be quality improvement. The role for the robotic-assisted approach as a minimally invasive option in reducing long-term opioid use is also an unanswered question**.** Previous studies reported that robotic-assisted surgery is associated with reduced postoperative pain [5, 6]. However, there is limited literature examining the potential benefit of the robotic approach in reducing short-term and long-term opioid use [7, 8], particularly for colectomies.

Using population-based insurance claims data, we aimed to evaluate long-term opioid use after colon resections and compare them by surgical approach. We hypothesized that (1) patients undergoing minimally invasive colon resection are less likely to fill long-term opioid prescriptions postoperatively compared to open surgery (OS) and (2) among MIS options, the robotic-assisted (RS) approach is associated with fewer opioid prescriptions filled than laparoscopic colectomy (LS).

## Materials and methods

The manuscript was prepared in accordance with the STROBE (STrengthening the Reporting of OBservational studies in Epidemiology) guideline for reporting observational cohort study.

### Data sources and study sample

This is a retrospective claims data analysis using the IBM® MarketScan® Research Database (MarketScan), a large dataset capturing linked and de-identified inpatient, outpatient and pharmacy services from a range of employer-provided health insurance companies [9]. The annual medical database report contains more than 40 million beneficiaries from approximately 350 payers in the US [9]. As an aggregated, de-identified, Health Insurance Portability and Accountability Act (HIPAA)-compliant database, retrospective analyses of MarketScan are considered exempt from informed consent and institutional review board approval.

**Fig. 1:**
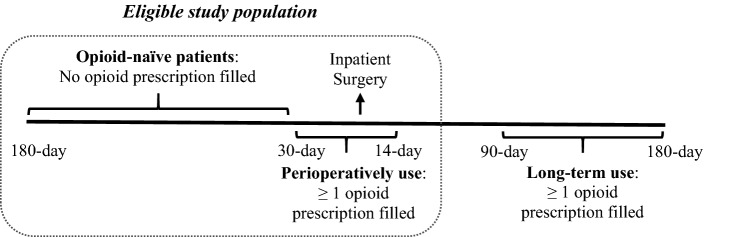
Study criteria and outcomes definition. Patients were included if they did not fill an opioid prescription 180 days before surgery (opioid-naïve) and had at least 1 opioid prescription filled 30 days before surgery to 14 days after discharge (perioperative use). The outcome of long-term opioid use was defined as at least 1 opioid prescription filled between 90 and 180 days after discharge; prolonged use was defined as 1 or more additional opioid prescription(s) filled between 180 and 365 days after discharge

The study population consisted of patients aged 18 years or older who underwent colon resection between 2013 and 2017. Procedures included sigmoidectomy and left or right hemicolectomy for benign and malignant indications. We used International Classification of Diseases and Related Health Problems, Ninth Revision (ICD-9) and Tenth Revision (ICD-10) codes to define the eligible colon resection cases and differentiate surgical approaches (Supplementary Table 1). For this study, eligible patients were those who did not fill an opioid prescription 30–180 days before surgery (opioid-naïve) and had at least one opioid prescription filled perioperatively (30 days before surgery to 14 days after discharge; Fig. 1). Patients receiving opioids within 30 days of surgery included prescriptions provided preoperatively for postoperative pain control. Inclusion of these patients as opioid-naïve is consistent with prior studies [1].

Because the dataset does not contain inpatient opioid use, we included only patients who had at least one opioid prescription filled perioperatively to capture all patients exposed to opioids after surgery. Study patients were required to be continuously enrolled with medical and pharmacy benefit coverage 180 days before to 180 days after colon resection to capture baseline comorbidities and evaluate complete health services utilization. We excluded patients who were opioid tolerant (defined as having opioid prescriptions filled between 30 and 180 days before admission for surgery), patients with an opioid use disorder or chronic pain diagnosis prior to surgery, patients with a prolonged hospital length of stay (LOS > 14 days; a surrogate for emergent or more complicated cases), multiple colon resections, any hospitalizations during the follow-up period, and patients with invalid opioid prescription data.

### Exposure variables

We identified surgical modality using ICD-9 and ICD-10 codes (Supplementary Table 1) and classified patients into OS, LS or RS groups. In the evaluation of MIS versus OS, LS and RS were merged into the MIS group.

### Outcome variables

Opioid prescriptions filled were identified using MarketScan pharmacy claims data. Perioperative opioid prescriptions were defined as any outpatient filled prescriptions from 30 days before admission to 14 days after discharge. Long-term opioid prescriptions were defined as at least 1 opioid prescription filled between 90 and 180 days after discharge. These definitions are consistent with prior studies and represent time frames in which uncomplicated postoperative recovery would be expected following colon surgery [3, 10]. For patients filling at least 1 opioid prescription perioperatively, we calculated the proportion of long-term opioid use (i.e., patients with ≥ 1 long-term opioid prescriptions filled 90–180 days after discharge) and categorized opioids as (1) ‘any opioids’ [obtained using the national drug code and linking the Marketscan drug claim data to a Center for Disease Control (CDC) opioids table], (2) controlled substance schedule II or III opioids [Schedule II or III opioids by Drug Enforcement Administration (DEA) classification in Marketscan dataset: https://www.dea.gov/drug-scheduling], and (3) high-dose opioids (≥ 50 Morphine Milligram Equivalents per day calculated based on prescriptions and analogous to morphine 7.5 mg q4 hours or oxycodone 5 mg q4 hours).

### Patient factors

Baseline sociodemographic and clinical characteristics included as potential confounders were age, sex, region, residential area metropolitan status, annual income level, insurance plan, year of surgery, presence of colorectal cancer and Charlson Comorbidity Index (CCI) scores. Insurance plans were classified into preferred provider organization (PPO), comprehensive insurance, health maintenance organization (HMO), point-of-service (POS) and other insurance plans. Using ICD-9/ICD-10 diagnosis codes, conditions known to increase the risk of opioid overuse were identified based on existing literature. These conditions were tobacco abuse or history, alcohol abuse or dependence, and mental health conditions (schizophrenia, mood disorders, anxiety, dissociative and somatoform problems, or depression) [10, 11]. Patient comorbidities and the above risk factors were evaluated at the index hospitalization and in the 180-day preoperative period.

### Statistical analysis

Propensity score matching (PSM) was used to balance baseline sociodemographic and clinical characteristics when comparing opioid prescriptions by surgical modality (MIS vs. OS and RS vs. LS). All patient factors described above and shown in Table 1 were included as covariates in the model performed to generate the propensity score based on prior studies and clinical relevance [3, 10, 11]. A separate category for “unknown” was created for infrequent variables with missing values**.** We used a logistic regression model to calculate the propensity scores that estimated the likelihood that a patient would receive either MIS versus OS or RS versus LS [12]. Then, 1:1 propensity score matching using a 5-to-1 digits greedy matching without replacement technique was used to create matched samples for analysis [13]. A standardized difference of < 0.1 was used to assess covariates balance achieved between the matched samples. Within each matched sample, logistic regression was used to assess the effect of the surgical approach on opioid prescriptions; covariates that were imbalanced after PSM were additionally adjusted in the regression model [14]. Odds ratios (OR) were calculated for opioid outcomes and considered statistically significant if the 95% confidence interval (CI) excludes the null value. In subgroup analyses, we separated malignant and non-malignant patients based on presence of diagnosis codes of colorectal cancer and repeated all analyses above respectively. Multivariate logistic regression was performed to assess risk factors for long-term opioid use. To understand the impact of LOS on opioid prescription fills, percentages of patients who filled any opioids were plotted based on colon resection inpatient LOS (≤ 3, 4, 5, 6, ≥ 7 days) as sensitivity analysis. All analyses were conducted using SAS version 9.4 (SAS Institute, Cary, NC, USA).

**Table 1:** Baseline sociodemographic characteristics before and after propensity score matching

	Before PSM			After PSM: OS vs. MIS			After PSM: LS vs. RS		
	OS (*n* = 5663)	LS (*n* = 8055)	RS (*n* = 1169)	OS (*n* = 5413)	MIS (*n* = 5413)	Std. diff	LS (*n* = 1169)	RS (*n* = 1169)	Std. diff
Age (years)						0.025			0.034
18–44	881 (15.6%)	1189 (14.8%)	140 (12.0%)	822 (15.2%)	865 (16.0%)		137 (11.7%)	140 (12.0%)	
45–54	1450 (25.6%)	2259 (28.0%)	348 (29.8%)	1404 (25.9%)	1374 (25.4%)		353 (30.2%)	348 (29.8%)	
55–64	2174 (38.4%)	3133 (38.9%)	464 (39.7%)	2096 (38.7%)	2106 (38.9%)		449 (38.4%)	464 (39.7%)	
Over 64	1158 (20.4%)	1474 (18.3%)	217 (18.6%)	1091 (20.2%)	1068 (19.7%)		230 (19.7%)	217 (18.6%)	
Male vs. female	2775 (49.0%)	3977 (49.4%)	599 (51.2%)	2637 (48.7%)	2643 (48.8%)	0.022	623 (53.3%)	599 (51.2%)	0.008
Income/year						0.036			0.049
< 40 k	4417 (78.0%)	6065 (75.3%)	881 (75.4%)	4200 (77.6%)	4271 (78.9%)		884 (75.6%)	881 (75.4%)	
40 k–49 k	785 (13.9%)	1250 (15.5%)	207 (17.7%)	765 (14.1%)	712 (13.2%)		195 (16.7%)	207 (17.7%)	
> 50 k	126 (2.2%)	194 (2.4%)	38 (3.3%)	123 (2.3%)	108 (2.0%)		37 (3.2%)	38 (3.3%)	
Unknown	335 (5.9%)	546 (6.8%)	43 (3.7%)	325 (6.0%)	322 (5.9%)		53 (4.5%)	43 (3.7%)	
Insurance plan						0.023			0.056
PPO	2950 (52.1%)	4316 (53.6%)	607 (51.9%)	2825 (52.2%)	2851 (52.7%)		610 (52.2%)	607 (51.9%)	
Comprehensive	787 (13.9%)	854 (10.6%)	164 (14.0%)	734 (13.6%)	703 (13.0%)		171 (14.6%)	164 (14.0%)	
HMO	569 (10.0%)	876 (10.9%)	121 (10.4%)	547 (10.1%)	563 (10.4%)		107 (9.2%)	121 (10.4%)	
POS	422 (7.5%)	617 (7.7%)	85 (7.3%)	405 (7.5%)	400 (7.4%)		88 (7.5%)	85 (7.3%)	
Others	853 (15.1%)	1241 (15.4%)	177 (15.1%)	822 (15.2%)	824 (15.2%)		173 (14.8%)	177 (15.1%)	
Unknown	82 (1.4%)	151 (1.9%)	15 (1.3%)	80 (1.5%)	72 (1.3%)		20 (1.7%)	15 (1.3%)	
Metro status						0.005			0.045
Metropolitan	1012 (17.9%)	1029 (12.8%)	131 (11.2%)	927 (17.1%)	919 (17.0%)		115 (9.8%)	131 (11.2%)	
Non-metropolitan	4586 (81.0%)	6935 (86.1%)	1029 (88.0%)	4425 (81.7%)	4431 (81.9%)		1045 (89.4%)	1029 (88.0%)	
Unknown	65 (1.1%)	91 (1.1%)	9 (0.8%)	61 (1.1%)	63 (1.2%)		9 (0.8%)	9 (0.8%)	
Region						0.034			0.030
Northeast	826 (14.6%)	1333 (16.5%)	193 (16.5%)	805 (14.9%)	754 (13.9%)		183 (15.7%)	193 (16.5%)	
North central	1577 (27.8%)	1886 (23.4%)	321 (27.5%)	1483 (27.4%)	1471 (27.2%)		334 (28.6%)	321 (27.5%)	
South	2482 (43.8%)	3702 (46.0%)	499 (42.7%)	2380 (44.0%)	2459 (45.4%)		498 (42.6%)	499 (42.7%)	
West	693 (12.2%)	1027 (12.7%)	147 (12.6%)	665 (12.3%)	650 (12.0%)		145 (12.4%)	147 (12.6%)	
Unknown	85 (1.5%)	107 (1.3%)	9 (0.8%)	80 (1.5%)	79 (1.5%)		9 (0.8%)	9 (0.8%)	
Benign vs. malignant	3544 (62.6%)	4803 (59.6%)	700 (59.9%)	3382 (62.5%)	3401 (62.8%)	− 0.007	722 (61.8%)	700 (59.9%)	0.039
Procedure type									
Sigmoidectomy	2505 (44.2%)	3343 (41.5%)	622 (53.2%)	2414 (44.6%)	2410 (44.5%)	− 0.001	648 (55.4%)	622 (53.2%)	− 0.045
Left colectomy	873 (15.4%)	848 (10.5%)	125 (10.7%)	788 (14.6%)	783 (14.5%)	− 0.003	125 (10.7%)	125 (10.7%)	0.000
Right colectomy	2406 (42.5%)	3998 (49.6%)	444 (38.0%)	2320 (42.9%)	2330 (43.0%)	0.004	421 (36.0%)	444 (38.0%)	0.041
Procedure year						0.025			0.060
2013	1107 (19.5%)	1635 (20.3%)	114 (9.8%)	1054 (19.5%)	1008 (18.6%)		111 (9.5%)	114 (9.8%)	
2014	1627 (28.7%)	2354 (29.2%)	236 (20.2%)	1535 (28.4%)	1528 (28.2%)		245 (21.0%)	236 (20.2%)	
2015	1323 (23.4%)	1838 (22.8%)	299 (25.6%)	1265 (23.4%)	1290 (23.8%)		320 (27.4%)	299 (25.6%)	
2016	1186 (20.9%)	1648 (20.5%)	370 (31.7%)	1156 (21.4%)	1166 (21.5%)		360 (30.8%)	370 (31.7%)	
2017	420 (7.4%)	580 (7.2%)	150 (12.8%)	403 (7.4%)	421 (7.8%)		133 (11.4%)	150 (12.8%)	
Tobacco abuse/Hx	948 (16.7%)	1300 (16.1%)	208 (17.8%)	891 (16.5%)	857 (15.8%)	− 0.017	199 (17.0%)	208 (17.8%)	0.020
Alcohol abuse/depend	144 (2.5%)	148 (1.8%)	15 (1.3%)	130 (2.4%)	130 (2.4%)	0.000	13 (1.1%)	15 (1.3%)	0.016
Mental health cond.	1043 (18.4%)	1405 (17.4%)	233 (19.9%)	1000 (18.5%)	983 (18.2%)	− 0.008	235 (20.1%)	233 (19.9%)	− 0.004
Charlson comorbidity						0.023			0.046
0	1454 (25.7%)	2220 (27.6%)	294 (25.1%)	1397 (25.8%)	1418 (26.2%)		302 (25.8%)	294 (25.1%)	
1–2	1701 (30.0%)	2737 (34.0%)	400 (34.2%)	1654 (30.6%)	1680 (31.0%)		393 (33.6%)	400 (34.2%)	
3–6	1179 (20.8%)	1888 (23.4%)	269 (23.0%)	1155 (21.3%)	1106 (20.4%)		284 (24.3%)	269 (23.0%)	
> 6	1329 (23.5%)	1210 (15.0%)	206 (17.6%)	1207 (22.3%)	1209 (22.3%)		190 (16.3%)	206 (17.6%)	

Standardized difference: values < 0.1 assumed to indicate negligible difference

*PSM* propensity score matching, *MIS* minimally invasive surgery, *OS* open surgery, *LS* laparoscopy, *RS* robotic-assisted surgery, *Std*. *diff* standardized difference, *PPO* preferred provider organization, *HMO* health maintenance organization, *POS* point-of-service

## Results

**Fig. 2:**
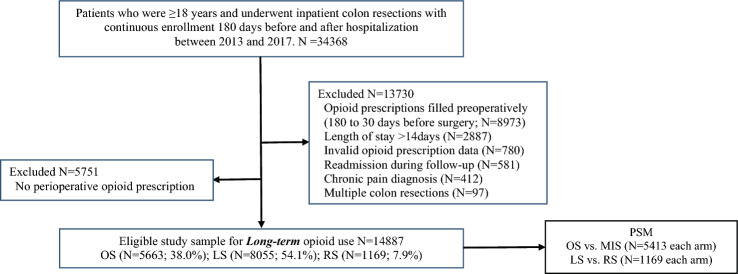
Study flowchart. *PSM* propensity score matching, *OS* open surgery, *MIS* minimally invasive surgery, *LS* laparoscopic surgery, *RS* robotic-assisted surgery

There were 34,368 continuously enrolled patients who underwent colon resection between 2013 and 2017. After excluding patients with prolonged inpatient LOS (> 14-day), chronic pain, multiple colon resections, any hospitalizations during 180-day follow-up, invalid opioid prescription data, and opioid prescriptions filled preoperatively (180–30 days before surgery), there were 20,638 opioid-naïve patients (Fig. 2). We further excluded 5751 patients (27.9%) who did not fill opioids perioperatively, and the final cohort consisted of 14,887 eligible patients for long-term use assessment: 5663 patients (38.0%) had OS, 8055 (54.1%) had LS and 1169 (7.9%) underwent RS. The distribution of benign and malignant disease was 39.2% malignant, 17.6% benign neoplasia, 29.4% diverticulitis, 5.5% inflammatory bowel disease, and 8.3% other diagnoses. After PSM, the final analysis consisted of 5,413 matched pairs in the MIS versus OS cohorts and 1169 matched pairs in the RS versus LS cohorts. Baseline sociodemographic and clinical characteristics were comparable in both matched sets (with standardized difference < 0.1; Table 1).

Among patients filling opioid prescriptions perioperatively, the incidence of long-term opioid use was 15.4% (2289 patients); 21.0% in OS, 12.2% in LS, and 10.9% in RS. After PSM, MIS significantly decreased long-term prescriptions of ‘any opioids’ when compared to OS (13.3% vs. 20.9%, *p* < 0.001), schedule II/III opioids (11.7% vs. 19.2%, *p* < 0.001), and high-dose opioids (4.3% vs. 7.7%, *p* < 0.001) from 90 to 180 days after discharge (Table 2). Among 1169 matched pairs of RS versus LS resections, RS significantly decreased long-term prescriptions of high-dose opioids (2.1% vs. 3.8%, *p* = 0.015), but no significant difference was observed in ‘any opioid’ use (11.0% vs. 12.8%) or schedule II/III opioid use (8.9% vs. 10.3%).

**Table 2:** Long-term opioid use in propensity score-matched cohorts, stratified by malignant status

All patients	PSM cohort: OS vs. MIS			PSM cohort: LS vs. RS		
	OS (*n* = 5413)	MIS (*n* = 5413)	*p* value	LS (*n* = 1169)	RS (*n* = 1169)	*p* value
Any opioid use	1130 (20.9)	719 (13.3)	< 0.001	138 (11.8)	127 (10.9)	0.473
Schedule II/III opioid use	1040 (19.2)	632 (11.7)	< 0.001	120 (10.3)	104 (8.9)	0.261
High-dose opioid use	414 (7.7)	233 (4.3)	< 0.001	44 (3.8)	24 (2.1)	0.015

Non-malignant patients	OS (*n* = 3349)	MIS (*n* = 3349)	*p* value	LS (*n* = 700)	RS (*n* = 700)	*p* value
Any opioid use	737 (22.0)	405 (12.1)	< .0001	88 (12.6)	58 (8.3)	0.012
Schedule II/III opioid use	682 (20.4)	349 (10.4)	< .0001	75 (10.7)	47 (6.7)	0.011
High-dose opioid use	278 (8.3)	131 (3.9)	< .0001	31 (4.4)	12 (1.7)	0.006

Malignant patients	OS (*n* = 2026)	MIS (*n* = 2026)	*p* value	LS (*n* = 469)	RS (*n* = 469)	*p* value
Any opioid use	381 (18.8)	311 (15.4)	0.004	60 (12.8)	69 (14.7)	0.277
Schedule II/III opioid use	346 (17.1)	276 (13.6)	0.002	57 (12.2)	27 (12.2)	0.845
High-dose opioid use	131 (6.5)	102 (5.0)	0.051	17 (3.6)	12 (2.6)	0.358

*PSM*, propensity score matching, *OS* open surgery, *MIS* minimally invasive surgery, *LS* laparoscopic surgery, *RS* robotic-assisted surgery, *Schedule II/III Opioid Use* controlled substance schedule II or III opioid use

Subgroup results for long-term opioid prescriptions between patients with malignant and non-malignant diagnoses are shown in Table 2. Among the patients receiving colectomy for non-malignant conditions, those who underwent MIS were less likely to fill long-term opioid prescriptions compared to OS patients in ‘any opioids’ (12.1% vs. 22.0%*, p* < 0.001), schedule II/III opioids (10.4% vs. 20.4%*, p* < 0.001) and high-dose opioids (3.9% vs. 8.3%*, p* < 0.001) categories. In the RS versus LS comparison, significantly lower rates of long-term opioid prescriptions filled were found in the RS than the LS non-malignant group for ‘any opioids’ (8.3% vs. 12.6%, *p* = 0.012), schedule II/III opioids (6.7 vs. 10.7%*, p* = 0.011), and high-dose opioids (1.7% vs. 4.4%, *p* = 0.006). MIS patients who had colon resections for colorectal malignancies had lower long-term fill rates of ‘any opioids’ (15.4% vs. 18.8%, *p* = 0.004), schedule II/III opioids (13.6% vs. 17.1%, *p* = 0.002) and high-dose opioids (5.0% vs. 6.5%, *p* = 0.051) when compared with OS. There were no significant differences between LS and RS malignant groups in any of the three long-term opioid measures.

Table 3 shows risk factors for long-term ‘any opioid’ prescription fills. Logistic regression revealed that significant risk factors are non-malignant disease (OR 1.20, 95% CI 1.06–1.35, *p* = 0.004), tobacco use (OR 1.28, 95% CI 1.14–1.44, *p* < 0.001), mental health conditions (OR 1.34, 95% CI 1.20–1.50, *p* < 0.001), higher Charlson comorbidities score (CCI > 6: OR, 2.06, 95% CI, 1.74–2.43, *p* < 0.001), and North Central (OR 1.23, 95% CI 1.03–1.47, *p* = 0.020), South (OR 1.36, 95% CI 1.14–1.61,* p* = 0.001), and West (OR 1.33, 95% CI 1.10–1.62, *p* = 0.004) regions. Conversely, laparoscopic (OR 0.58, 95% CI 0.53–0.64, *p* < 0.001) and robotic-assisted (OR 0.48, 95% CI 0.40–0.59, *p* < 0.001) approaches, age > 64 years (OR 0.81, 95% CI 0.68–0.97, *p* = 0.023), and right colectomy (OR 0.61, 95% CI 0.43–0.87, *p* = 0.006) were associated with lower risk of long-term opioid use.

**Table 3:** Multivariate logistic regression model for long-term opioid use

	Adjusted odds ratio	95%confidence limits		*p* value
Surgical approach				
Open	Ref			
Laparoscopic	0.58	0.53	0.64	< 0.001
Robotic-assisted	0.48	0.40	0.59	< 0.001
Age				
18–44	Ref			
45–54	0.89	0.77	1.03	0.121
55–64	0.92	0.80	1.05	0.218
Over 64	0.81	0.68	0.97	0.023
Male vs. female	1.01	0.92	1.11	0.788
Income/year				
< 40 k	Ref			
40 k–49 k	0.87	0.74	1.01	0.073
> 50 k	0.77	0.55	1.08	0.129
Unknown	0.98	0.77	1.25	0.867
Insurance plan				
PPO	Ref			
Comprehensive	1.05	0.89	1.25	0.541
HMO	0.95	0.81	1.12	0.559
POS	1.10	0.93	1.31	0.266
Others	1.05	0.92	1.20	0.477
Unknown	0.90	0.62	1.31	0.577
Metro status				
Metropolitan	Ref			
Non-metropolitan	1.06	0.93	1.20	0.368
Unknown	0.74	0.30	1.80	0.506
Region				
Northeast	Ref			
North central	1.23	1.03	1.47	0.020
South	1.36	1.14	1.61	0.001
West	1.33	1.10	1.62	0.004
Unknown	1.83	0.84	4.00	0.131
Benign vs. malignant	1.20	1.06	1.35	0.004
Procedure type				
Sigmoidectomy, yes	0.91	0.65	1.28	0.601
Left colectomy, yes	0.98	0.70	1.36	0.895
Right colectomy, yes	0.61	0.43	0.87	0.006
Procedure year				
2013	Ref			
2014	0.98	0.85	1.12	0.709
2015	0.92	0.80	1.06	0.241
2016	0.90	0.78	1.04	0.145
2017	0.86	0.70	1.05	0.130
Tobacco abuse/history	1.28	1.14	1.44	< 0.001
Alcohol abuse/depend	1.31	0.98	1.74	0.068
Mental health cond.	1.34	1.20	1.50	< 0.001
Charlson comorbidity				
0	Ref			
1–2	1.03	0.91	1.17	0.627
3–6	1.15	0.99	1.35	0.072
> 6	2.06	1.74	2.43	< 0.001

*OS* open surgery, *LS* laparoscopy, *RS* robotic-assisted surgery, *PPO* preferred provider organization, *HMO* health maintenance organization, *POS* point-of-service

**Fig. 3:**
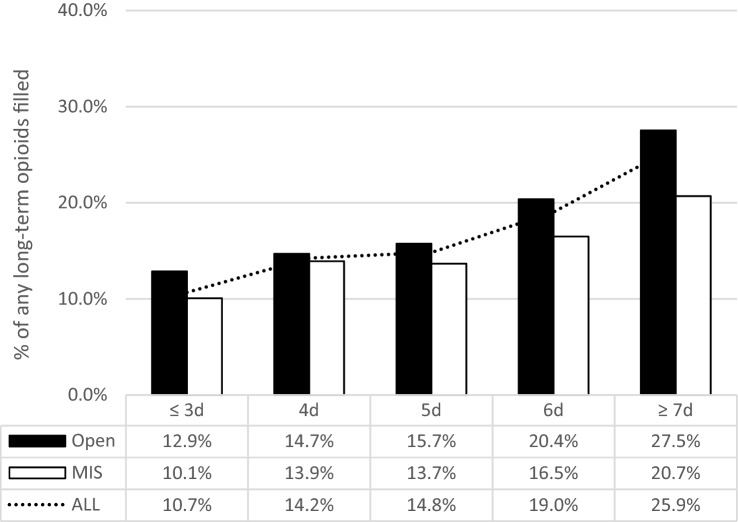
Percentages of patients who filled any opioids from 90 to 180 days after discharge by duration of the hospital stay in propensity score-matched cohorts. *MIS* minimally invasive surgery, *d* day

Figure 3 demonstrates the filling rates of ‘any opioids’ by duration of hospital LOS comparing MIS to OS surgical approaches. Longer LOS led to higher long-term opioids fill rates and the trend was found in both OS (≥ 7 days vs. ≤ 3 days: 27.5% vs. 12.9%) and MIS groups (≥ 7 days vs. ≤ 3 days: 20.7% vs. 10.1%).

## Discussion

Persistent opioid use after surgery is common and patients undergoing colorectal surgery are at higher risk compared to other procedures [3, 15]. This large database analysis shows that among opioid naïve patients, MIS was associated with a significantly lower risk of opioid filled prescriptions at 90–180 days after colon resection compared with open. Additionally, high-dose long-term opioid filled prescriptions are significantly less for RS than for LS. These differences seem to be most apparent following operations for non-malignant diseases, while patients with cancer show fewer significant differences in opioid use based on surgical approach. Additional risk factors for long-term opioid use include younger age, tobacco and alcohol abuse, mental health conditions, and Charlson comorbidities score ≥ 3. Patients undergoing right colectomy were less likely to develop long-term opioid use.

Revision of prescribing practices that include opioid reduction strategies have been devised to address the opioid crisis by limiting the amount of unused opioids available for community diversion and decreasing long-term opioid dependence. It is thought that the first opioid prescription to 100 patients results in one heroin user, and almost 75% of heroin users start with prescription medications as their introduction to opioids [16]. The ability to decrease opioids prescribed at discharge will decrease the amounts of unused opioids available for community diversion. Colorectal surgery is a risk factor for long-term opioid use in opioid-naïve patients [3]. Our study showed that the incidence of long-term opioid use is 13.5% to 21.2% and is consistent with other reports [15, 17]. Though there are several studies evaluating the risk for long-term opioid use for multiple procedures, there are few comparing the MIS and open approaches to colorectal surgery [18–20]. A single institution analysis of 2173 abdominopelvic procedures with intestinal resection revealed that 91% of patients were discharged on opioids, 4% were taking opioids 30 days after discharge, and 1% were still on opioids at 90 days. The odds of prolonged opioid use were significantly decreased with the minimally invasive approach compared to open [19]. This study was a single institution analysis in contrast to our large database analysis focused on colectomies. Others have confirmed open surgery as a risk factor for persistent opioid use after gastrointestinal surgery [18]. Our database analysis adds to previous studies by suggesting that the MIS approach to colectomy is an opioid reduction strategy and that the RS approach may be considered where expertise and resources are available, especially for non-malignant diseases.

High-dose opioid prescription fills were less for RS than LS. Subgroup PSM and logistic regression analysis in our study suggests that the significant difference in prescription fills between LS and RS for non-malignant diagnoses is not apparent for malignant conditions. The reasons for the apparent robotic-assisted advantage was not obvious from this analysis. Differences in enhanced recovery protocols and variation in surgeon expertise may be confounding factors. It is also possible that the intracorporeal anastomosis is utilized in a higher percentage of robotic-assisted than laparoscopic colectomies, resulting in less mesenteric traction and shorter incisions with less pain and opioid use [21–23]. The intracorporeal anastomosis is commonly used for robotic-assisted right colectomy and may also explain why right colectomy is associated with less long-term opioid use in this study [21, 23].

There is a paucity of literature comparing pain and opioid use in patients undergoing colectomy for malignant versus non-malignant colorectal diseases. One study showed significantly more postoperative opioid use for non-malignant compared to malignant conditions [24]. However, this study is an inpatient opioid use analysis in contrast to our study that is focused on long-term opioid use. Other studies without comparisons to non-malignant conditions have suggested that up to 15% of patients having oncologic resection develop persistent opioid use [25, 26]. A large, national insurance claims analysis of patients having colectomy for cancer showed that 8–17% of patients had persistent opioid use, with patients receiving chemotherapy at the higher end of the range [25]. Unlike our study, there was no comparison to a non-malignant group. It may be that patients and prescribers of patients with painful non-malignant diseases like diverticulitis and Crohn’s disease have higher opioid expectations after surgery and discharge, thereby contributing to long-term opioid use. Patients with Crohn’s disease often receive chronic opioids for abdominal pain though opioid therapy has not been shown to ameliorate chronic pain [27]. The non-malignant versus malignant colorectal diagnosis differences in long-term opioid use require further study.

Similar to our study, other researchers have identified younger age, tobacco and alcohol abuse, and mental health conditions as risk factors for long-term opioid use [10, 20, 24]. Another large insurance claims database analysis showed that new persistent opioid use > 90 days after colectomy among opioid-naïve patients was about 10%, and was not due to surgical pain but rather tobacco, alcohol, substance abuse, mood, anxiety, and pain disorders [3]. These investigators included a wide variety of minor and major surgical procedures, while our study was focused on colectomies alone. Others have also described American Society of Anesthesiologists (ASA) Classification and comorbidities as risk factors for long-term opioid use [18, 28].

Sensitivity analysis showed that longer hospital LOS led to higher long-term opioid use rates. There are a couple of possible explanations for this observation. It may be that longer LOS is a proxy for more complications resulting in higher opioid use following discharge. Others have shown that higher inpatient opioid use is associated with postoperative complications and longer LOS [24]. It is also possible that we need to devise more effective strategies weaning patients off opioids as inpatients rather than waiting until after discharge. The longer patients are receiving opioids, the greater is the risk for dependence with one study showing that opioid dependence was significantly associated with prolonged LOS in patients undergoing lumbar fusion [29].

This study is limited by retrospective design that may not account for unknown confounders. Though MIS and RS were associated with less long-term opioid use relative to OS and LS, respectively, causality cannot be inferred. We were unable to control for factors that impact surgical approach choice and other operative decision making. We were unable to control for race/ethnicity because the database does not include that variable. However, we included several predictors not available in many databases such as insurance type and geographic region. Only opioid-naïve patients not taking opioids more than 30 days before surgery were included in this study, because our focus was on comparing MIS and open surgical approaches in the opioid-naïve patient population. Therefore, our results are not generalizable to patients with a history of chronic opioid use. While we included patients receiving opioids within 30 days of surgery defined as opioid naïve based on previous studies, future studies with different definitions may consider this a covariate misclassification. Because about 9–25% of patients have a history of preoperative opioid use, the long-term opioid use values may have been higher had we included the chronic opioid use patient population [28, 30]. The dataset does not include information on inpatient opioid use, and we were unable to control for enhanced recovery pain management programs that include opioid sparing analgesics, transversus abdominis plane blocks for MIS, epidural analgesia for open colectomies, and other opioid reduction methods in the inpatient setting.

Patients with a hospital stay longer than 14 days were excluded from the analyses, assuming that prolonged LOS is a proxy for emergent or more complicated cases. We wanted our study to be a comparison of relatively uncomplicated operative approaches. If we included patients with hospital stay > 14 days, our results may have been even less favorable for the open complicated cases with longer length of stay. Though our sensitivity analysis showed increase in opioid use with increasing LOS for both open and MIS groups, including 14 days in exclusion criteria may be considered arbitrary. It has been determined that less than 50% of pain management discharge strategies currently employ non-opioid medications [31]. We were unable to control for patient-reported outcomes like postoperative complications, pain control, and patient satisfaction. This study does not include uninsured or underinsured patients, and our results may not be generalizable to these patient populations. Finally, while prescription claims data may be the best marker available to assess opioid prescriptions readily available in the community as well as long-term medication use (short of contacting patients), the data does not necessarily identify patient adherence.

With the development of better opioid prescribing practices, algorithms, and monitoring schemes, it is important for health care providers to weigh options that increase the effectiveness of opioid reduction and decrease long-term opioid use [32]. Choosing an MIS option and RS for some colorectal operations is a modifiable factor that may contribute to less long-term opioid use [19]. We recognize that patients continue to use opioids long after surgical pain has resolved, that long-term opioid use may not be related to prolonged postoperative pain as much as emotional pain, and that affective distress and post-discharge opioid use is proportional to the quantity prescribed [3, 28, 33]. Further research is required to determine the reasons for differences in long-term opioid use among colectomy surgical approaches and how to better manage contributing factors.

## Conclusion

The minimally invasive approach to colon resection for opioid naïve patients is associated with a significant reduction in long-term opioid use when compared to the open approach. Increasing minimally invasive training efforts and adoption may prove to be an opioid reduction strategy. Additionally, the robotic-assisted surgical approach reduced the use of high-dose opioids compared to laparoscopic colectomy and may be an option for non-malignant diseases where resources and expertise are available.

## Supplementary Information

Below is the link to the electronic supplementary material.

Supplementary file1 (DOCX 13 KB)
