# Supplementary material for: A national database propensity score-matched comparison of minimally invasive and open colectomy for long-term opioid use
Source: Surg Endosc. 2021 Feb 10;36(1):701–10. doi: 10.1007/s00464-021-08338-9 (PMC8741658; doi:10.1007/s00464-021-08338-9)
Supplement: Supplementary file 1 — Supplementary file1 (DOCX 13 KB) [file 464_2021_8338_MOESM1_ESM.docx]

Supplementary Table 1: Procedure and indication codes used for eligibility

| **Measure** | **Grouping** | **Code type** | **Code** |
| --- | --- | --- | --- |
| Sigmoidectomy | Laparoscopic | ICD-9-PCS | 17.36 |
|  |  | ICD-10-PCS | 0DTN4ZZ |
|  | Open | ICD-9-PCS | 45.76 |
|  |  | ICD-10-PCS | 0DTN0ZZ |
| Left colectomy | Laparoscopic | ICD-9-PCS | 17.35 |
|  |  | ICD-10-PCS | 0DTM4ZZ, 0DTG4ZZ |
|  | Open | ICD-9-PCS | 45.75 |
|  |  | ICD-10-PCS | 0DTM0ZZ, 0DTG0ZZ |
| Right colectomy | Laparoscopic | ICD-9-PCS | 17.32, 17.33 |
|  |  | ICD-10-PCS | 0DTF4ZZ, 0DTH4ZZ, 0DTK4ZZ |
|  | Open | ICD-9-PCS | 45.72, 45.73 |
|  |  | ICD-10-PCS | 0DTF0ZZ, 0DTH0ZZ,0DTK0ZZ |
| Robotic surgery | N/A | CPT | S2900 |
|  |  | ICD-9-PCS | 17.4x |
|  |  | ICD-10-PCS | 8E0**CZ |
| Malignant (colorectal) | N/A | ICD-9-CM | 15.30, 15.31, 15.32, 15.33, 15.34, 15.35, 15.36, 15.37, 15.38, 1539, 19.75, 15.40, 15.41 |
|  |  | ICD-10-CM | C180, C181, C182, C183, C184, C185, C186, C187, C188, C189, C785, C19, C20 |
